# Experience and Expectations of Ovarian Cancer Patients in Australia

**DOI:** 10.1155/2018/7863520

**Published:** 2018-03-07

**Authors:** Catherine M. Holliday, Maria Morte, Josephine M. Byrne, Anne T. Holliday

**Affiliations:** Centre for Community-Driven Research, Ultimo, NSW, Australia

## Abstract

Some of the most significant advances in ovarian cancer treatment have been those that result in improvements in progression-free survival (PFS); however there is little research to understand the value that patients place on accessing therapies that result in PFS as a clinical outcome related to survivorship. This study therefore aimed to understand the experience and expectations of women with ovarian cancer in Australia in relation to quality of life (QoL) and treatment options. An online survey collected demographic information and 13 investigator-derived structured interview questions were developed to understand the experience of women with ovarian cancer, their understanding of terminology associated with their condition, and expectations of future treatment. This study demonstrated that ovarian cancer patients equate PFS with being in remission and that patients expect QoL during that time to be good to excellent. Women in this study described excellent QoL as feeling positive and happy and not worrying about cancer, feeling fit and healthy without side effects, and being able to live life as they did before their diagnosis, including the absence of fear of progression or recurrence. It is therefore suggested that there is a positive relationship between PFS and QoL. While it is difficult to quantify QoL and further research is needed, the results of this study suggest that the minimum time that women with ovarian cancer expect in relation to treatments that result in PFS is approximately six months. In the absence of this information, decision-makers are left to make assumptions about the value women place on access to therapeutics that increase PFS, which for this type of cancer is an important aspect of survivorship.

## 1. Introduction

There are three main types of ovarian cancer including germ cell and sex cord-stromal tumors which account for approximately 10% of all ovarian cancers, and the most common type, epithelial ovarian cancer, accounting for approximately 90% of all cases [1]. Within the epithelial group, high-grade serous ovarian cancer (HGSOC) is the most common histological subtype, an advanced stage cancer, and the most common cause of ovarian cancer deaths [2].

There has however been no appreciable improvement in the overall survival for women with advanced epithelial ovarian cancer over the last 20 years. Although the majority of patients initially respond to platinum based chemotherapy, most will relapse and the most significant advances in advanced ovarian cancer treatments have been those that result in improvements in PFS [3, 4]. There is however little research to understand the expectation that patients have of quality of life (QoL) and the value they place on accessing therapeutics that result in PFS as a clinical outcome, which is an important aspect of survivorship.

We therefore designed a study that aimed to understand the experience and expectations of women with ovarian cancer in the context of the Australian health system in relation to QoL and treatment options.

## 2. Methods and Materials

An online survey was used to collect demographic information and 13 investigator-derived structured interview questions were developed to understand the experience of women with ovarian cancer in the Australian health system, their understanding of the terminology associated with their condition, and expectations of future treatment. 

### 2.1. Participants

To be eligible for the study, women needed to have been diagnosed with ovarian cancer in Australia, be 18 years of age or older, be able to speak English, and be able to give consent to participate in the study. Participants were recruited via email and Facebook through the networks of the Centre for Community-Driven Research and Ovarian Cancer Australia, both of which are nonprofit patient organisations in Australia and women self-selected to participate in the study.

### 2.2. Data Collection

Demographic data for the online survey was collected using Survey Monkey (Survey Monkey Inc., Palo Alto, California, USA, https://www.surveymonkey.com). Participants completed the survey between July and August 2015.

Interviews were conducted by telephone. The interviews were recorded and transcribed verbatim. Identifying names and locations were not included in the transcription. All transcripts were checked against the original recording for quality assurance.

### 2.3. Analysis

A content analysis was conducted using conventional content analysis methodology to identify major themes from structured interviews [5]. Conventional content analysis is a process of identifying original themes from raw data without the influence of preconceived categories.

Text from the interviews was imported into NVivo 8 (QSR International) and read line-by-line to develop initial themes and definitions which were registered in NVivo. A theme was identified by developing a description of each event, experience and/or, expectation by a participant. The minimum coded unit was a sentence; however there were also paragraphs and phrases that were coded as a unit. For a theme to be included, it needed to have occurred more than three times across all interviews.

A second researcher verified the initial codes and definitions, and the text was coded until full agreement was reached with both researchers using the process of consensual validation [6]. The frequency of coded sentences and phrases was calculated using total number of coded phrases within a specific question.

## 3. Results

### 3.1. Demographics

40 women completed the online survey in July 2015, and 35 participants completed the structured interviews. The five women that did not complete the structured interview withdrew from this part of the study due to illness. The majority of participants had a primary ovarian tumor (*n* = 31, 12.5%), of which 21 had HGSOC (52.5%). Demographics of all participants are available in Table 1.

### 3.2. Structured Interview Results

The first interview question was an open question designed to make the participant at ease and set the context for the remainder of the interview. As demographic information was collected in the online questionnaire, an analysis of question 1 was not conducted.

Question 2 asked participants to define the terms mild side effects, moderate side effects, and severe side effects. The collective definition of mild side effects was feeling slight discomfort, having mild or no nausea, being able to conduct activities of daily living, and being able to manage discomfort with medication. The most common definition (*n* = 24; 50.0%) was in relation to “Slight discomfort, irritation, or annoyance; side effect is not really an issue or intrusive.” The least coded theme was “Issues that are manageable with medication” (*n* = 3, 6.3%).

Moderate side effects were collectively described as some nausea/gastric disturbances, fatigue, needing assistance with some activities of daily living, general discomfort, and needing to seek some medical attention. The two most commonly cited definitions were “Able to do some activities of daily living but need assistance at times” (*n* = 14, 26.9%) and being “Not comfortable, feeling generally unwell, and/or issues that linger” (*n* = 15, 28.8%). “Tiredness, fatigue, or fogginess” was the next most frequent theme (*n* = 10, 19.2%) with the least cited themes being “Some nausea and/or gastric disturbance” (*n* = 7, 13.5%) and the “Need to seek medical attention or manageable with medication” (*n* = 6, 11.5%).

Severe side effects were described as extreme vomiting and nausea, neuropathy, needing assistance with activities of daily living, debilitating fatigue and pain, needing medical assistance or hospitalization, and constant issues that may not be able to be resolved. 62 phrases or sentences were coded and the most frequently coded theme was “Fatigue requiring full bed rest throughout the day, debilitating” (*n* = 15, 24.2%) followed by assistance required with activities of daily living, issues that impact family (*n* = 13, 21.0%). Neuropathy was the least coded theme (*n* = 3, 4.8%).

Question 3 asked participants to define the terms poor, good, and excellent quality of life. 154 phrases were coded within this question of which 59 were coded for poor, 44 for good, and 51 for excellent quality of life. Participants collectively described poor quality of life as feeling uncomfortable most of the time, not being able to function, requiring bed rest throughout the day, and feeling depressed and not enjoying many aspects of life. “Not being able to function normally or do activities of daily living without help” was the most commonly cited theme (*n* = 30, 50.8%), followed by “Feeling unwell or in pain most or uncomfortable most or all of the time” (*n* = 14, 23.7%).

Good quality of life was described as feeling positive and happy, feeling some discomfort from time-to-time, being able to make choices and continue with activities of daily living, and feeling well. The ability to make choices and continue normally with activities of daily living was the most frequently coded theme (*n* = 23, 52.3%), followed by “Some pain or discomfort and/or the odd bad day; needing rest from time-to-time” (*n* = 12, 27.3%) and “Feeling positive and happy, having good friends and family; finding pleasure or joy in activities/work” (*n* = 9, 20.5%).

Excellent quality of life was described as feeling positive and happy and not worrying about cancer (*n* = 16, 31.4%), feeling fit and healthy without side effects (*n* = 10, 19.6%), and being able to live life as they did before their diagnosis (*n* = 25, 49.0%). While participants noted that excellent quality of life would include the psychological aspect of not having to worry about recurrence, they also commented that, in their situation, this was not realistic.

Question 4 asked participants what “being on treatment” meant to them. 53 phrases were coded across four themes. The most commonly cited response described being on treatment as traditional aspects of treatment such as chemotherapy, radiation therapy, or surgery (*n* = 24, 45.3%) while 20 participants (37.7%) felt that any type of medical therapy or medical intervention would be considered treatment. Five participants associated being on treatment with actively seeing their oncologist for checkups and tests (9.4%). The least less common theme was the concept of a clinical trial and treatment (*n* = 4, 7.5%).

Question 5 asked participants “What is your expectation of quality of life are during treatment.” 32 phrases were coded and the collective definitions given by participants in question 3 of this interview were used to code responses to question 5. Responses to this question indicated that participants primarily expected a poor quality of life (*n* = 25, 78.1%) with a minority of participants expecting a good quality of life (*n* = 7, 21.9%).

In question 6 participants were asked what factors or influences were important in their treatment decision-making. 57 phrases were coded within this question. The most common factors or influences were discussing treatment options with their partner or spouse and/or family along with their treating clinician (*n* = 16, 28.1%). 11 participants (19.3%) described their treating clinician as the primary decision-maker and noted that this was sometimes without being presented with options or discussion. Participants also described the importance of the whole medical team and/or their GP as part of their decision-making process (*n* = 9, 15.8%) and balancing side effects, quality of life, and impact on family (*n* = 9, 15.8%). The least frequent themes were related to the importance of having choice and/or trusting their clinician (*n* = 6, 10.5%) while some participants described making treatment decisions primarily on their own as an individual decision (*n* = 6, 10.5%).

Question 7 asked participants what their definition of being in remission was. 46 phrases were coded with the most common definition of remission described as having no evidence of disease and/or a low CA 125 and/or no signs or symptoms of cancer (*n* = 23, 50.0%). 10 participants (21.7%) stated that they were unsure and/or have never been told or felt like they were in remission, while seven participants (15.2%) described being in remission as finishing treatment and/or not having to go to doctors' appointments. The feeling that there was a lingering sense of anxiety and fear of recurrence during remission and/or knowing that cancer is still there but not active was also noted by six participants (13.0%).

Question 8 asked about expectations of quality of life during remission. A comparison of results is available in Table 2. 42 phrases were coded and the collective definitions given by participants in question 3 of this interview were used to code responses to question 8. 23 participants (54.8%) described the expectation of an excellent quality of life and nine participants (21.4%) described that their expectation was to have good quality of life. 10 participants (23.8%) also noted that the reality of quality of life is different to their expectations and/or noted that the possibility of recurrence lingers regardless of quality of life.

There was a relationship between expectations during remission and the number of lines of chemotherapy that a participant had been through. As the number of lines of chemotherapy increased, the expectation of quality of life during remission decreased. Fifteen participants that had been through one line of chemotherapy expected an excellent quality of life compared with six who had been through two lines of chemotherapy and two participants who had been through three lines of chemotherapy.

Question 9 asked participants whether they would prefer to continue under surveillance or on maintenance therapy and why. 72 phrases or sentences were coded within this question. Seven participants (*n* = 7, 10.8%) stated that they were not sure what the terms meant. There was little difference between preferences with 16 (24.6%) participants stating that they preferred maintenance therapy and 18 (27.7%) preferring surveillance. Participants who had been through two lines of chemotherapy had a lower preference for surveillance therapy (11.8%) compared to the overall group (27.7%).

The rationale for those that stated a preference for maintenance therapy was primarily to remain active in managing their cancer and/or maintain a sense of control (*n* = 10/16, 62.5%), with four of the 16 participants (25.0%) noting that their preference for maintenance therapy depended on side effects and/or impact on QoL. The primary rationale for the 18 participants who preferred surveillance was to avoid additional treatment or medical attention (*n* = 10/18, 55.6%). Participants that stated a preference for surveillance also noted that this is based on their current situation and/or that no alternative was available and/or no alternative was offered (*n* = 7/18, 38.9%).

In question 10, participants were asked whether they had heard of the term “progression-free survival.” 35 phrases or sentences were recorded within this question. The majority of participants had not heard of the term (*n* = 29, 60.0%). Nine participants had heard of it but did not know its meaning (25.7%). Five participants (14.3%) had heard of the term and offered their own definition that related to being in remission. Similarly, those that had not heard of the term or did not know its meaning were offered a definition and primarily translated that to being in remission.

Questions 11 and 12 asked “If you were offered a treatment that increased the length of time before your cancer progressed, how long would you expect your cancer to be stable for it to be worth taking?” Participants were asked to consider whether there would be a difference if the treatment had mild side effects (question 11) or moderate to severe side effects (question 12).

**Figure 1:**
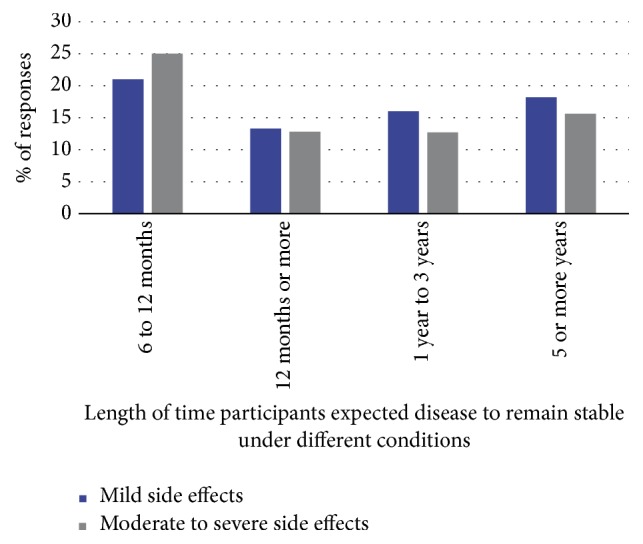
*Expectations of disease progression.* Participants were asked what length of time they expected their disease to remain stable with treatment under different conditions (mild side effects or moderate to severe side effects). Overall the most commonly stated expectation was that the cancer would be stable between six and 12 months.

There were no significant differences between the length of time the participants expected their cancer to be stable with either mild or moderate to severe side effects, and the most commonly stated expectation between both groups was that their cancer would be stable between six and 12 months (Figure 1).

In relation to treatments with mild side effects, 7 participants (18.4%) stated that they would take therapy regardless of the amount of time that the cancer remained stable, while 4 participants (12.5%) stated that they would not take any treatment with moderate to severe side effects.

Participants stated that this was a very difficult question to answer. Five participants (13.2%) were unable to answer question 11 and seven participants (21.9%) were unable to answer question 12.

In question 13 participants were asked whether their decision-making process changed when they were making decisions about treatment during remission. 35 sentences or phrases were coded. 23 participants (65.7%) stated that their decision-making process did not change. 12 participants (34.3%) stated that it did as they placed more focus on quality of life and less invasive treatment options.

## 4. Discussion

### 4.1. Ovarian Cancer Patients' Understanding of Terminology

The results of this study demonstrate that there are some terms that patients with ovarian cancer are familiar with; however there are others that are not well understood (see Table 3). Within the study, participants were able to confidently describe the terms “quality of life,” “side effects,” and “treatment,” while terms such as “remission,” “progression-free survival,” “surveillance,” and “maintenance therapy” were less understood.

The importance of clarity around the term remission is particularly important with ovarian cancer patients as approximately 70% of women will experience an eventual recurrence [7]. Fear of recurrence during remission is also a significant concern among ovarian cancer patients and one that to date has not been sufficiently addressed [8]. Of particular concern to women is the anxiety associated with fear of recurrence. One study reported that 61% of women have an increased level of anxiety when tested for CA-125 [9] while a second study reported that 62.5% of women have a high to extreme level of anxiety at checkups [10]. It follows then that easing fear of recurrence during remission may increase QoL, which is also consistent with the results of this study where women noted that excellent QoL during remission included not having to worry about cancer recurrence.

The majority of women within our study had not heard of the term “progression-free survival”; however when prompted to describe what they thought it meant, most women equated PFS to being in remission. During remission, participants' expectations of QoL were good to excellent. This suggests that while the term PFS is not well understood, it is valued in the context of remission and is associated with QoL.

PFS is becoming increasingly important as a clinical trial endpoint in ovarian cancer and there are significant discussions in relation to how this translates into decisions about access to affordable therapeutics [11]. Given the focus that governments in particular place on patient engagement in the decision-making process, it is important to acknowledge that where discussions are driven by the term “progression-free survival,” patients may not understand the term and therefore may not be able to engage in a meaningful way. To address this, community engagement efforts in health policy should focus on questions that encourage the community members to articulate what they value and/or expect from treatments, rather than being driven by technical terminology.

### 4.2. Ovarian Cancer Patients' Expectations

The participants in our study demonstrated a clear difference in expectations of quality of life during treatment compared to times of remission. During times of treatment, there was a general expectation that QoL would be poor to good; however during times of remission, there was a general expectation that life would be good to excellent. There was also a relationship between expectations during remission and disease progression, specifically that as disease progressed, the expectation of quality of life during remission decreased.

This is consistent with a large study that observed QoL of over 3000 patients across different stages of disease. The study by Ferrell et al. (2005) found a significant variance in QoL according to stage; QoL for those with early stage disease had better QoL compared to those with late stage disease where QoL was largely influenced by fear of recurrence [12]. There are however limitations in comparing QoL between early and late stage disease as the majority of women are diagnosed with late stage ovarian cancer [13, 14] and quantifying the value that women place on QoL, particularly in times of remission, is challenging given that many QoL studies are conducted during times of treatment [15].

A recent online study by Coleman et al. (2013) asked ovarian cancer patients to quantify the minimum benefit in PFS that a therapy would have for it to be valuable. The majority of participants selected 5 or more months, and when asked to tradeoff overall survival with toxicity compared to PFS without toxicity, 44% of participants chose the latter while 37% found neither acceptable [16]. In our study we found that the minimum length of time that participants stated a treatment would be valuable with both mild or moderate to severe side effects was 6 months. It is important to note the difference in terminology used in both studies; the Coleman study used the term “toxicity,” whereas our study referred to “side effects.” This may in part account for the difference in results.

### 4.3. Decision-Making and Patient Preference

It is acknowledged that the decision-making process regarding treatment is complex. Stage of disease, type of diagnosis (primary or recurring), management of symptoms, and quality of life are all contributing factors that influence treatment decisions between patients and clinicians [17].

The results of our study are aligned with those of a previous study by Ziebland et al. (2006) that investigated different approaches to decision-making with ovarian cancer patients. The authors observed that the majority of women with primary ovarian cancer diagnoses were dependent on their doctors to recommend treatment options with the best possible outcome of a cure, as they knew little about the disease or treatments available [18]. A second study demonstrated that women with recurring disease are more likely to be involved in decision-making process but still felt overwhelmed by the decision-making process itself [19]. In general, the more information that is presented to women, particularly in relation to how to manage possible side effects and/or improve psychological autonomy, the more confident they are in their decision-making [20], and involvement in decision-making is associated with better quality of life [21]. Nevertheless, as observed in our study and the study by Ziebland et al. (2006), some women stated that they could not always recall being actively involved in decisions; several women felt that there were no real decisions to make or had their physicians decide on treatment [18].

As noted previously, fear of recurrence or metastasis is a significant concern for ovarian cancer patients and is a significant consideration in relation to decision-making [18]. The need to present patients with available options has been documented in a number of studies, particularly in relation to decision-satisfaction and reducing anxiety [22–25]. An important observation in our study was that during times of remission, there was little difference between preferences between maintenance therapy and surveillance; however participants that stated a preference for surveillance also noted that this is based on their current situation and/or that no alternative was available and/or no alternative was offered.

We were unable to identify any other studies that specifically asked women about preference for surveillance or maintenance therapy. There are however studies that investigated QoL with participants undergoing treatment or surveillance. An Australian study investigated the impact on QoL when chemotherapy is administered to women with platinum-resistant, recurrent disease [26]. The authors reported that QoL neither increased or decreased; that is, the overall QoL of participants was maintained. In a Canadian study, QoL was measured using the European Organization for Research and Treatment of Cancer Quality of Life Questionnaires (EORTC QLQ-C30 and OV28) as well as an investigator-developed questionnaire [27]. A subfactor analysis of the 102 participants indicated that there were no significant variations in overall global quality of life and functional scales between those who were undergoing surveillance (46%) and those with active disease (54%). The results of our study and previous studies therefore suggest that the decision to commence maintenance therapy or surveillance therapy has less impact on QoL than being informed and presented with the option to choose.

### 4.4. Limitations

The study size of 40 participants is relatively small; however, given the intensity of the structured interviews, scientific merit can be met in small study populations, even those that do not reach traditional conditions for statistical power, and legitimate sample sizes can be made for cost and feasibility reasons [28], as was the case in this study.

It is also important to note that with any study that relies on self-selection, there is always the potential for bias. The main risk is that the participants who choose to be involved in a study may not represent the target population, which may be viewed as a limitation to this study.

## 5. Conclusion

In the absence of patient insights such as those reported in this study, decision-makers are left to make assumptions about the value women place on access to therapeutics and the values that they would like to see included in the decision-making process.

This study demonstrated that ovarian cancer patients equate PFS with being in remission and that patients expect QoL during that time to be good to excellent. Women in this study described excellent QoL as feeling positive and happy and not worrying about cancer, feeling fit and healthy without side effects, and being able to live life as they did before their diagnosis, including the absence of fear of progression or recurrence. It is therefore suggested that there is a strong relationship between PFS and QoL. While it is difficult to quantify QoL and further research is needed, the results of this study suggest that the minimum time that women with ovarian cancer value, in relation to PFS and therefore QoL, is approximately six months.

While there were few differences between treatment decision-making during times of treatment and times of remission, there was a distinct difference in the expectation and experience of QoL during the different time points. This suggests that additional mechanisms and options are needed to reduce the gap between the current experience of women with ovarian cancer and what they expect from treatment options.

Finally, the terminologies used to describe things such as PFS, maintenance therapy, and surveillance, all of which are increasingly important in both clinical, research, and policy decision-making, are not well understood by patients. It is important to acknowledge this and ensure that appropriate language and mechanism are used so that their experience can be valued in research and in the decision-making process.

## Figures and Tables

**Table 1:** Participant demographics.

Demographic	*n* = 40	%
*Age*		
35 to 44	5	12.5%
45 to 54	10	25.0%
55 to 64	10	25.0%
65 to 74	12	30.0%
75 or older	3	7.5%
*Geographic location within Australia*		
NSW	15	37.5%
VIC	12	30.0%
QLD	7	17.5%
WA	6	15.0%
*Level of education*		
Less than high school degree	1	2.5%
High school degree or equivalent	5	12.5%
Some university but no degree	12	30.0%
Bachelor degree	11	27.5%
Associate degree	5	12.5%
Graduate degree	6	15.0%
*Year of diagnosis*		
2008	2	5.0%
2009	1	2.5%
2010	4	10.0%
2011	1	2.5%
2012	12	30.0%
2013	13	32.5%
2014	7	17.5%
*Primary tumour site*		
Ovary	31	77.5%
Fallopian tube	8	20.0%
Peritoneum	1	2.5%
*Histology*		
Epithelial	34	85.0%
(i) Serous: high grade	21	52.5%
(ii) Serous: low grade	3	7.5%
(iii) Clear cell	3	7.5%
(iv) Endometrioid	3	7.5%
(v) N/A or unknown	4	10.0%
Sex cord stromal	1	2.5%
Unknown	5	12.5%
*BRCA status*		
Negative	20	50.5%
Positive	7	17.5%
Unknown	13	32.5%
*Lines of chemotherapy completed*		
0	1	2.5%
1	20	50.0%
2	12	30.0%
3	5	12.5%
4	1	2.5%
5	1	2.5%
*Current treatment status*		
Maintenance therapy after primary treatment	3	7.5%
Surveillance after primary treatment	16	40.0%
Maintenance therapy postrecurrence	5	12.5%
Surveillance postrecurrence	4	10.0%
Recurrence, with no treatment	2	5.0%
Recurrence, with treatment	9	22.5%
Palliative	1	2.5%

**Table 2:** Comparison of quality of life during treatment compared to remission.

	QoL during remission	QoL during treatment
Poor QoL	0%	78.1%
Good QoL	21.4%	21.9%
Excellent QoL	54.8%	0%

**Table 3:** Definitions of words used by women in the explanation of this illness and the treatments used.

Terminology	Definitions of words used by women in the explanation of this illness and the treatments used
Mild side effects	feeling slight discomfort, having mild or no nausea, being able to conduct activities of daily living, and being able to manage discomfort with medication.
Moderate side effects	Some nausea/gastric disturbances, fatigue, needing assistance with some activities of daily living, general discomfort, and needing to seek some medical attention.
Severe side effects	Extreme vomiting and nausea, neuropathy, needing assistance with activities of daily living, debilitating fatigue and pain, needing medical assistance or hospitalization, and constant issues that may not be able to be resolved.
Poor quality of life	Feeling uncomfortable most of the time, not being able to function, requiring bed rest throughout the day, and feeling depressed and not enjoying many aspects of life.
Good quality of life	Feeling positive and happy, feeling some discomfort from time to time, being able to make choices and continue with activities of daily living, and feeling well.
Excellent quality of life	Feeling positive and happy and not worrying about cancer, feeling fit and healthy without side effects, and being able to live life as they did before their diagnosis.
Being on treatment	Traditional aspects of treatment such as chemotherapy and radiation therapy or surgery or any type of medical therapy or medical intervention
Remission	Having no evidence of disease and/or a low CA 125 and/or no signs or symptoms of cancer (noting that some participants also noted that they were unsure and/or have never been told or felt like they were in remission)
Progression-free survival	The majority of participants had not heard of the term and did not know what it meant. Where a definition was offered, it primarily related to being in remission.

## Abbreviations

HGSOC: High-grade serous ovarian cancer
PFS: Progression-free survival
QoL: Quality of life.
